# Anthropometric Profiling, Somatotype Differentiation, and Methodological Discrepancies in Body Fat Estimation Across Combat Sports: A Comparative Multi-Equation Analysis

**DOI:** 10.3390/jfmk11040435

**Published:** 2026-10-20

**Authors:** Armando Monterrosa-Quintero, Alex Ojeda-Aravena, Adriana Guzmán-Sanchez, Boryi A. Becerra-Patiño, Julian Mauricio Soto Morcote, Jorge Alberto Charry Fernandez, Adrián De la Rosa

**Affiliations:** 1 Altius Performance Laboratory, Physical Education and Sports Program, Universidad Surcolombiana, Neiva 410010, Colombia; joalchafe14@hotmail.com; 2 Departamento de Ciencias de la Actividad Física, Universidad de Los Lagos, Osorno 5290000, Chile; 3 School of Kinesiology, Universidad Bernardo O’Higgins, Santiago 8370993, Chile; 4 Program of Nursing, Faculty of Health and Basic Sciences, Fundación Universitaria Navarra (UNINAVARRA), Neiva 410010, Colombia; adrianaguzman2511@gmail.com; 5 Faculty of Physical Education, National Pedagogical University, Bogotá 110221, Colombia; babecerrap@pedagogica.edu.co; 6 Faculty of Physical Culture, Sport and Recreation, Universidad Santo Tomás, Tunja 150001, Colombia; julian.sotom@usantoto.edu.co; 7 Body, Physical Activity and Sport Study Group (GECAFD), Sports Department, Universidad Industrial de Santander, Bucaramanga 680002, Colombia; adrian.rosa@uv.es; 8 Freshage Research Group, Department of Physiology, Faculty of Medicine, University of Valencia, CIBERFES, Fundación Investigación Hospital Clínico Universitario/INCLIVA, 46010 Valencia, Spain

**Keywords:** martial arts, muscle fractionation, phantom proportionality, method comparison, concordance correlation coefficient, multivariate ordination

## Abstract

**Objectives:** To evaluate kinanthropometric profiles, somatotypes, Phantom *z*-scores, and methodological discrepancies among nine body fat percentage (%BF) equations relative to the Katch & McArdle comparator equation across combat sports. **Methods:** Sixty-seven elite male athletes (Brazilian Jiu-Jitsu: *n* = 17, Muay Thai: *n* = 16, Wrestling: *n* = 16, Taekwondo: *n* = 18) underwent complete ISAK profiling. Differences across disciplines were assessed via ANOVA, while inter-equation agreement was evaluated using repeated-measures ANOVA, Bland–Altman metrics, Lin’s concordance correlation coefficient (*ρ_c_*), and principal component analysis (PCA). **Results:** Striking and grappling disciplines exhibited distinct morphological profiles, with Taekwondo displaying an ecto-mesomorphic habitus versus the endo-mesomorphic profile of grappling combat sports. Substantial systematic differences and proportional biases were observed across %BF equations (*p* < 0.001). Withers demonstrated the highest agreement with the comparator equation, whereas Durnin & Womersley showed a marked positive bias. PCA ordination (accounting for 71.78% of variance) successfully separated morphological robusticity from linearity. **Conclusions:** Anthropometric %BF equations produce substantial systematic discrepancies and should not be considered interchangeable when monitoring combat athletes. External validation against an independent criterion method is required before establishing absolute body fat values.

## 1. Introduction

Athletic performance is characterized by highly demanding competition with narrow margins in expression of capabilities, leading to successful or unsuccessful outcomes [1]. Physical preparation is a determining factor in athletic success [2], alongside body composition (BC) [3,4]. This is because fat percentage and muscle mass are significantly associated with a better expression of technical-tactical skills in combat sports [5,6]. Combat sports are characterized by a high manifestation of physical, physiological, nutritional, biomechanical, and BC characteristics [7], and depending on physiological demands, these can be characterized as intermittent high-intensity and high-impact sports [8,9,10].

In these sports, the evaluation of BC is of vital importance because it allows monitoring nutritional adequacy, health, body development, and the effects of sports training [11]. Thus, surface anthropometry is a fast and easily accessible technique that allows the evaluation of BC, providing information on the skeletal, muscle, fat mass, and residual components [12]. To this end, the International Society for the Advancement of Kineanthropometry (ISAK) standardized quality protocols on anthropometric measurements that define parameters to evaluate the different anthropometric indicators [13]. This method seeks to measure superficial body dimensions, such as skinfolds, diameters, lengths, and circumferences to provide information on morphological status [14,15], and these circumferences, in turn, provide information about body geometry that is influenced by adipose and muscle tissue, which can hinder or favor the manifestation of a performance [16]. Similarly, skinfold measurements provide valuable information about subcutaneous adipose tissue [17,18], which is a key factor influencing endurance and mobility [19]. On the other hand, the somatotype has been used as a classification of athletes’ morphological characteristics to identify their dimensions and proportions [4].

In sports, the z-score calculated using the Phantom strategy is a crucial tool for identifying talent and monitoring athletes’development. It allows the detection of significant deviations in anthropometric measurements, such as size, shape, or BC, which can confer biomechanical advantages or indicate areas for improvement. For example, a high z-score in muscle mass could indicate an aptitude for strength sports, while extreme values in other variables could suggest risks or needs for intervention. Additionally, the z-score plays a key role in longitudinal monitoring during training programs, as it helps differentiate changes resulting from the intervention (e.g., muscle mass gains from training) from those associated with natural growth, particularly in young athletes [20].

Achieving an adequate assessment of BC is necessary to identify the most decisive aspects in different sports and to establish reference values for comparing the BC of athletes in the same sport [21,22], which is why it is necessary to continue developing studies that help to understand these specific features related to the sports context. Thus, the use of the BC assessment is required to develop a systematic and rigorous follow-up of athletes who practice modalities with weight categories such as combat sports [23]. Likewise, specialized literature has confirmed that adequate BC can be associated with a significant increase in cardiorespiratory strength and capacity [24,25].

Sports such as Jiu Jitsu, Muay Thai, Wrestling, and Taekwondo are classified as weight-sensitive sports, because athletes compete in weight categories and must undergo extreme diets characterized by dehydration processes to reduce body mass (BM) [26], especially the percentage of body fat (%BF) to enable athletes to move to lower weight categories [23]. Thus, systematic measurements of BC are necessary within sports planning [27]. This is because the monitoring of weight and BC serves as a control variable, because generally in the preseason in combat sports athletes are assessed by using the measurement of skinfolds to estimate %BF, which is later used as an indicator to calculate the minimum wrestling weight that is part of a wrestling certification program [28]. In this sense, there are different equations used based on the prediction of skinfolds and different BC methodologies that have been investigated in combat sports [29,30,31,32].

Despite the critical role of body composition and somatotype in combat disciplines governed by weight categories [23,26], comprehensive investigations simultaneously contrasting kinanthropometric proportionality and muscle fractionation remain scarce. While individual studies have examined isolated characteristics in specific martial arts [2,28] and highlighted the severe limitations of general-population formulas in wrestlers and grapplers [29,30], a comparative multi-equation and proportional framework across diverse striking and grappling modalities specifically building upon previous athletic validation approaches such as those of Monterrosa et al. [33] for body fat estimation equations and Withers et al. [34] for athletic reference models is lacking.

Therefore, this study aimed to: (1) determine the morphological, somatotypic, and proportional Phantom z-score differentiation across four competitive combat sports (Brazilian Jiu-Jitsu, Muay Thai, Wrestling, and Taekwondo); (2) evaluate the extent of systematic divergence and concordance across nine widely used anthropometric body fat prediction equations relative to the Katch & McArdle [35] and Withers [34] comparator equations; and (3) elucidate the multivariate morpho-functional ordination of combat athlete s through unsupervised principal component analysis. We hypothesized that grappling and striking disciplines would exhibit divergent structural adaptations, particularly in upper-limb muscularity and linearity, and that general-population equations would demonstrate unacceptable systematic discrepancies compared to athletic-specific models.

## 2. Materials and Methods

### 2.1. Experimental Design

**Figure 1:**
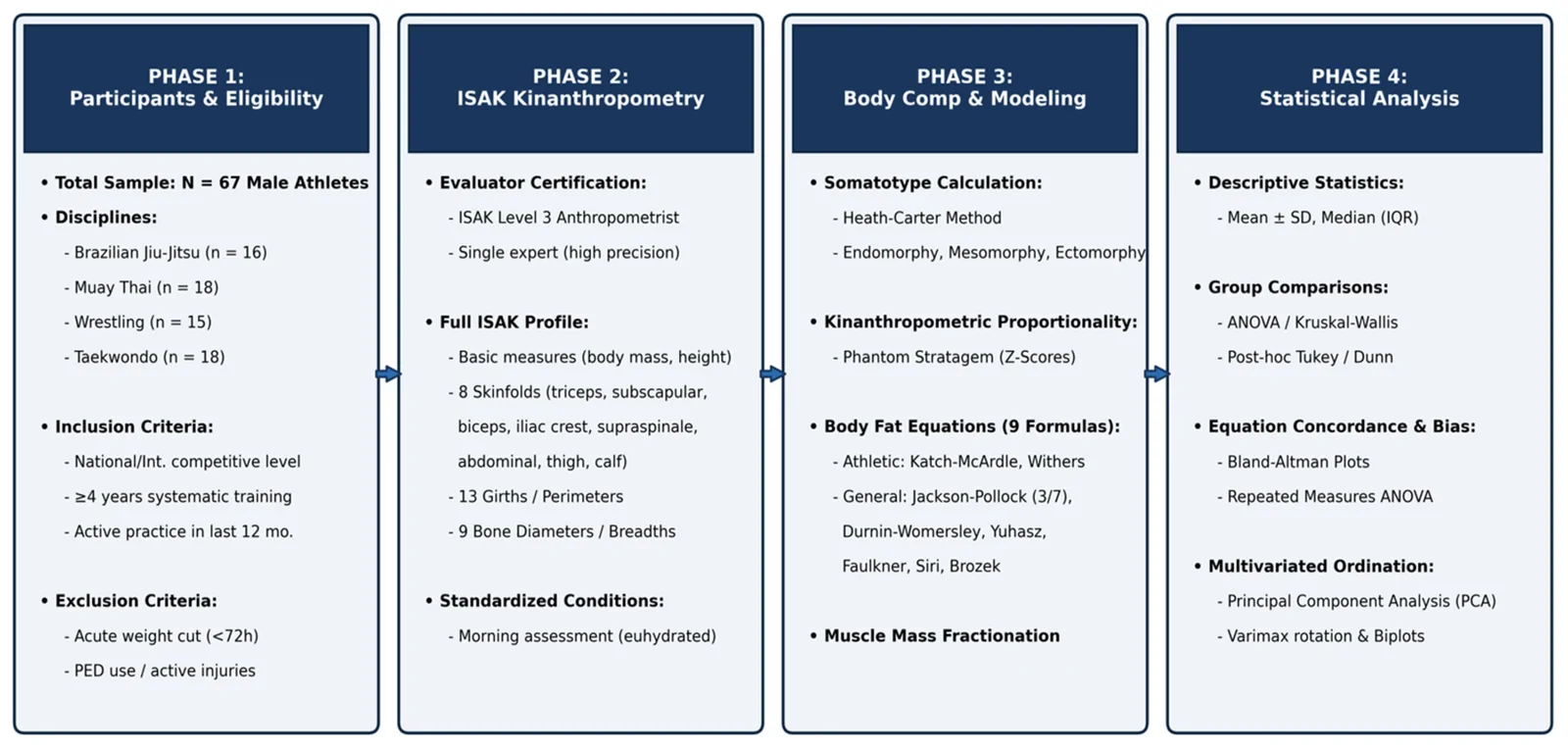
Flowchart of the experimental design and methodological workflow across the study phases.

This study utilized an observational, cross-sectional, and comparative multi-group design to evaluate kinanthropometric profiles, somatotype distribution, Phantom proportionality z-scores, and methodological divergences in body fat estimation across four distinct combat sports (Brazilian Jiu-Jitsu, Muay Thai, Wrestling, and Taekwondo). Data collection was conducted under standardized laboratory and environmental conditions to minimize physiological and technical fluctuations (Figure 1).

### 2.2. Participants and Eligibility Criteria

Sixty-seven (*N* = 67) elite male athletes representing four competitive combat disciplines were recruited from high-performance training centers and academies in Brazil and Colombia, distributed as follows: Brazilian Jiu-Jitsu (*n* = 17), Muay Thai (*n* = 15), Wrestling (*n* = 19), and Taekwondo (*n* = 16).

Inclusion criteria: Athletes were required to meet the following conditions: (1) active competitive status and regular participation in national or international championships; (2) a minimum of four years of systematic training and practical experience in their respective combat discipline; (3) absence of any musculoskeletal, metabolic, or disabling injuries at the time of evaluation; and (4) consistent training engagement during the preceding twelve months.

Exclusion criteria: Participants were excluded if they had: (1) recent rapid weight-cutting or acute dehydration protocols within 72 h prior to the evaluation; (2) a history of performance-enhancing substance use; or (3) incomplete anthropometric profiles or withdrawal of voluntary consent.

All participants were active national- and international-level competitors currently in their regular preparation phase, engaging in a mean of 14.2 ± 3.5 h per week of discipline-specific technical-tactical training and structured strength and conditioning (5–6 sessions per week), with an overall training background of 7.8 ± 2.9 years. Athletes competed predominantly across light-to-middleweight categories (<70 kg to <85 kg), avoiding heavy weight-class extremes to preserve cohort homogeneity. Geographically, Jiu-Jitsu and Muay Thai cohorts were evaluated at specialized high-performance academies in Brazil, whereas Wrestling and Taekwondo athletes were assessed at national sports technification centers in Colombia. To guarantee absolute standardization across sites, all assessments across both countries were conducted under strictly identical laboratory settings (07:00–10:00 a.m., 24 °C controlled temperature, post-overnight fast) utilizing the same calibrated ISAK-certified instrumentation and executed exclusively by the same ISAK Level 3 anthropometrist.

Sample size determination was conditioned by the strict inclusion criteria for elite-level status and the specific access to competitive cohorts across high-performance training centers in Brazil and Colombia. Rather than being determined by an a priori equal-allocation restriction, the final distribution per discipline reflects active population availability during the evaluation window. From a methodological standpoint, sample sizes ranging from 15 to 20 observations per group comply with established statistical rules of thumb for univariate and multivariate group comparisons [36,37,38], ensuring robustness in ANOVA *F*-tests and satisfactory Central Limit Theorem properties under homogeneous variances. Furthermore, no sample loss or post-recruitment attrition occurred; all recruited athletes who met eligibility criteria successfully completed the ISAK profiling protocol. Following observational study reporting guidelines (STROBE/CONSORT extensions for cross-sectional designs), this sample size provided sufficient statistical power (*β* > 0.80, *α* = 0.05) to detect large effect sizes (*η*^2^ ≥ 0.20) across morphological and somatotypic comparisons.

### 2.3. Procedures

Data collection took place in the morning hours (between 07:00 and 10:00), ensuring that participants had not engaged in strenuous physical exercise or training during the 24 h preceding the anthropometric assessments.

### 2.4. Anthropometric Evaluation

Anthropometric measurements were carried out after verifying that the athletes had complied with this 24-h restriction, to minimize variations in BC. The anthropometrist conducted all evaluations in an air-conditioned environment at a constant temperature of 24 °C. A level 3 anthropometrist certified by the International Society of the Advancement of Kinanthropometric (ISAK) performed the measurements, strictly following its standardized protocol (complete profile). The procedure began with the marking of anatomical reference points using a dermographic pencil, according to the sites defined by ISAK to measure skin folds, bone diameters, perimeters, and lengths. All measurements were taken on the right side of the participants’ bodies, in duplicate, with a third measurement in case of discrepancies greater than the allowable technical error (5% for skinfolds and 1% for other measurements).

The following calibrated instruments were used: Seca 216 stadiometer (1 mm sensitivity) (Hamburg, Germany) to measure height, and Tanita FitScan BC-545F (Tanita Corporation, Tokyo, Japan) scale (100 g sensitivity), which the researchers used exclusively to determine total body mass. All body composition metrics and relative adiposity percentages were calculated using standardized anthropometric prediction equations, while Basal Metabolic Rate (BMR; kcal/day) was estimated mathematically using the revised Harris–Benedict equation [39] based on anthropometric parameters (body mass, stature, and age), strictly avoiding proprietary bioimpedance-derived (BIA) metabolic estimates. Additional equipment included an anthropometric bench with dimensions of 40 cm in height, 50 cm in width, and 35 cm in depth for sitting height, pachymeter with long and short diameters, metal tape measure (1 mm sensitivity), clinical caliper (1 mm sensitivity), and segmometer, all from Cescorf^®^ (Porto Alegre, Brazil).

This study was classified as minimal risk in accordance with Resolution 8430 of 1993 from the Ministry of Health and Social Protection of Colombia. The research was conducted in accordance with the Declaration of Helsinki and approved by the Ethics, Bioethics and Scientific Integrity Committee (CEBIC) of Universidad Santo Tomás Seccional Tunja (Protocol code: DII-2025-001). Prior to testing, all participants were informed of the benefits and risks of the study, gave their voluntary consent, and signed a written informed consent form.

### 2.5. Comparison of Anthropometric Variables (Z-Score)

For the standardization and comparison of anthropometric variables, the phantom methodology (z-score) proposed by Ross & Wilson (1974) [40] calculates the z-score for each measurement. This procedure allows adjustment of the body dimensions to a theoretical reference model of 170.18 cm in height, eliminating the influence of body size and facilitating comparisons between individuals of different sizes and contexts. The raw values of each variable were transformed using the following equation:

$$Z=\frac{V{\left(\frac{170.18}{h}\right)}^{d}-p}{s}$$

where “*V*” is the value obtained for the variable under study, “170.18” is the constant of proportionality for height in the Phantom model, “*h*” is subject’s height, “*d*” is the dimensional exponent, “*p*” is the Phantom mean value for variable “*V*”, and “*s*” is the standard deviation for variable studied in the Phantom model [41]. This model ensures that the observed differences reflect true variations in morphology, independent of height, and has been widely validated for comparative analysis in sports and non-sports populations.

### 2.6. Somatotype

The Heath–Carter anthropometric somatotype (1967 method) [42] was calculated to determine the somatotypic profile and body build of the athletes. This standardized anthropometric method evaluates physique through three fundamental components: endomorphy (relative adiposity derived from skinfolds), mesomorphy (musculoskeletal robustness scaled by height, bone diameters, and corrected girths), and ectomorphy (relative linearity expressed by the height-weight ratio, height divided by the cube root of body mass). Component ratings were computed using standard anthropometric formulas based on bilateral measurements of skinfolds (triceps, subscapular, supraspinale, medial calf), bone breadths (humerus and femur biepicondylar diameters), and girths (flexed arm and calf circumferences) [42].

### 2.7. Body Fat Prediction Equations and Methodological Characteristics

To evaluate inter-method divergence and systematic agreement in body fat estimation, nine widely utilized anthropometric prediction equations were modeled. The selection, categorization, and implementation of these equations followed the methodological framework previously established by Monterrosa et al. [33], ensuring standardized application across athletic cohorts. These models encompass diverse mathematical approaches, including formulas estimating body density (*Db*) converted via Siri [43] or Brožek et al. equations [44], as well as equations predicting relative body fat percentage (%BF) directly from specific skinfold combinations. The mathematical characteristics, specific anatomical sites, and original validation populations of the evaluated equations are detailed below (Table 1).

### 2.8. Statistical Analysis

Data normality and homoscedasticity were verified using the Shapiro–Wilk test and Levene’s test, respectively. Continuous variables were expressed as mean ± standard deviation (SD) or median and interquartile range (IQR), as appropriate. Differences across the four combat disciplines (Brazilian Jiu-Jitsu, Muay Thai, Wrestling, and Taekwondo) were evaluated using one-way analysis of variance (ANOVA). When statistical significance was observed, Tukey’s Honestly Significant Difference (HSD) post-hoc test was applied for pairwise multiple comparisons. Effect sizes were quantified via partial eta squared (*η*^2^), interpreted as small (≥0.01), medium (≥0.06), or large (≥0.14).

To evaluate the comparative performance of the nine body fat prediction equations, statistical procedures were organized into four distinct analytical pillars according to their specific purpose: (A) Systematic differences: Overall inter-equation divergence across the cohort was evaluated using repeated-measures ANOVA with the prediction model defined as a within-subject factor (assessing sphericity via Mauchly’s test and applying Greenhouse–Geisser corrections when appropriate), complemented by the non-parametric Friedman test. (B) Agreement: Pairwise agreement relative to the Katch & McArdle [35] comparator equation was evaluated using Bland–Altman analysis, reporting mean bias and 95% limits of agreement (LoA = bias ± 1.96 × SD_diff) alongside their corresponding 95% confidence intervals (95% CIs) for both the mean bias and limits of agreement to account for sampling uncertainty. (C) Concordance: Absolute alignment and precision relative to the comparator model were quantified using Lin’s Concordance Correlation Coefficient (ρc) with its corresponding 95% confidence interval. (D) Proportional bias: Dependency of inter-method divergence on overall adiposity magnitude was formally examined via linear regression modeling the pairwise differences against the mean of the two methods. Finally, an unsupervised principal component analysis (PCA) based on standardized z-scores was conducted as an exploratory multivariate ordination and dimensionality reduction technique to evaluate the underlying morphological and functional relationships of the athletes across somatotype dimensions, skeletal robustness, regional muscularity, relative adiposity, and metabolic turnover. All statistical analyses and graphical visualizations were performed in R (version 4.5.2; R Foundation for Statistical Computing, Vienna, Austria) using the tidyverse, rstatix, DescTools, FactoMineR, and factoextra packages. Statistical significance was established a priori at α = 0.05.

For detailed data supporting the findings of this study, including complete anthropometric and body composition spreadsheets, please refer to the Data Availability Statement.

## 3. Results

Descriptive data of the anthropometric characteristics, including basic measurements, skinfold thicknesses, circumferences, and body diameters, are presented in Table 2.

Combat discipline significantly influenced anthropometric variability, accounting for large effects in muscularity, age, and body dimensions (Table 2). The strongest effect emerged in upper-limb hypertrophy, where the sport explained 56% and 54% of the total variance in flexed and relaxed arm girths, respectively (*η*^2^ = 0.56 and 0.54, *p* < 0.001); post-hoc contrasts confirmed that Taekwondo athletes possessed significantly smaller arm perimeters than the other three modalities, while Jiu-Jitsu fighters also exceeded wrestlers. Modality accounted for 28% of the variance in waist girth (*p* < 0.001), with narrower midsections in Taekwondo compared to Jiu-Jitsu and Muay Thai, and in wrestlers relative to Jiu-Jitsu. Discipline accounted for 43% of the variance in age (*p* < 0.001), separating older Jiu-Jitsu and Muay Thai competitors from younger Wrestling and Taekwondo athletes. The sport factor further explained 20% of the variance in stature (*p* = 0.003), uniquely distinguishing wrestlers as shorter than all other cohorts, and 17% in body mass (*p* = 0.007), isolating heavier Jiu-Jitsu practitioners from lighter Taekwondo fighters. Conversely, skeletal breadths, maximal calf girth, and skinfold thicknesses were statistically invariant across disciplines (*η*^2^ ≤ 0.13), showing no meaningful sport-dependent differentiation.

Table 3 presents the mean and standard deviation values of the anthropometric characteristics standardized using the Phantom method in athletes from different combat disciplines (Jiu-Jitsu, Muay Thai, Wrestling, and Taekwondo). The comparative analysis reveals statistically significant differences among the modalities in key variables such as body mass (z-Body Mass), girths (relaxed and flexed arm, waist, and hip), and breadths (humerus and femur, *p* < 0.05), reflecting the specific morphological profiles demanded by each combat sport. Conversely, most of the normalized skinfold thicknesses showed no significant differences among the evaluated groups.

Combat discipline significantly influenced muscularity, somatotype distribution, and general body build, with large effect sizes across key morphological parameters (Table 4). The most pronounced effect in the study emerged in upper-limb muscularity: modality accounted for 58% of the total variance in arm muscle area (η^2^ = 0.58, *p* < 0.001), with post-hoc pairwise contrasts confirming a clear hierarchical gradient in which Brazilian Jiu-Jitsu exceeded Muay Thai and Wrestling, while Taekwondo athletes displayed substantially lower muscular cross-sectional areas than those of athletes in all other modalities. A corresponding large effect appeared in body mass index (24% explained variance, *p* < 0.001), uniquely isolating Taekwondo fighters, whose body mass index was lower than that of fighters in the other three disciplines.

Somatotypic profiling revealed distinct structural adaptations driven by competitive discipline. The sport factor explained 30% of the variance in mesomorphy (η^2^ = 0.30, *p* < 0.001) and 26% in ectomorphy (η^2^ = 0.26, *p* < 0.001), segregating Taekwondo athletes characterized by markedly lower musculoskeletal development and a higher linearity/slenderness profile from the robust, mesomorphic profiles of Jiu-Jitsu, Wrestling, and Muay Thai competitors. Conversely, endomorphy remained uniform across cohorts (6% explained variance, *p* = 0.255), aligning with the lack of significant differences in total subcutaneous adiposity (Σ6 and Σ8 skinfolds, *p* > 0.05).

Regarding relative adiposity estimation, significant discipline effects emerged in three specific skinfold equations: Petroski (19% explained variance, *p* = 0.004), Jackson (18%, *p* = 0.005), and Guedes (15%, *p* = 0.016). In each case, post-hoc contrasts identified lower estimated body fat percentages in Taekwondo athletes relative to Jiu-Jitsu and Muay Thai cohorts. The remaining six equations (Faulkner, Withers, Durnin, Katch, Slaughter, and Yuhasz) as well as arm adipose area showed non-significant inter-group variation (η^2^ ≤ 0.12, *p* > 0.05), underscoring that global adiposity levels were relatively preserved across these combat sports. Table 4 presents the results of the equations applied, as well as the BC and somatotype comparisons among the four martial arts.

**Figure 2:**
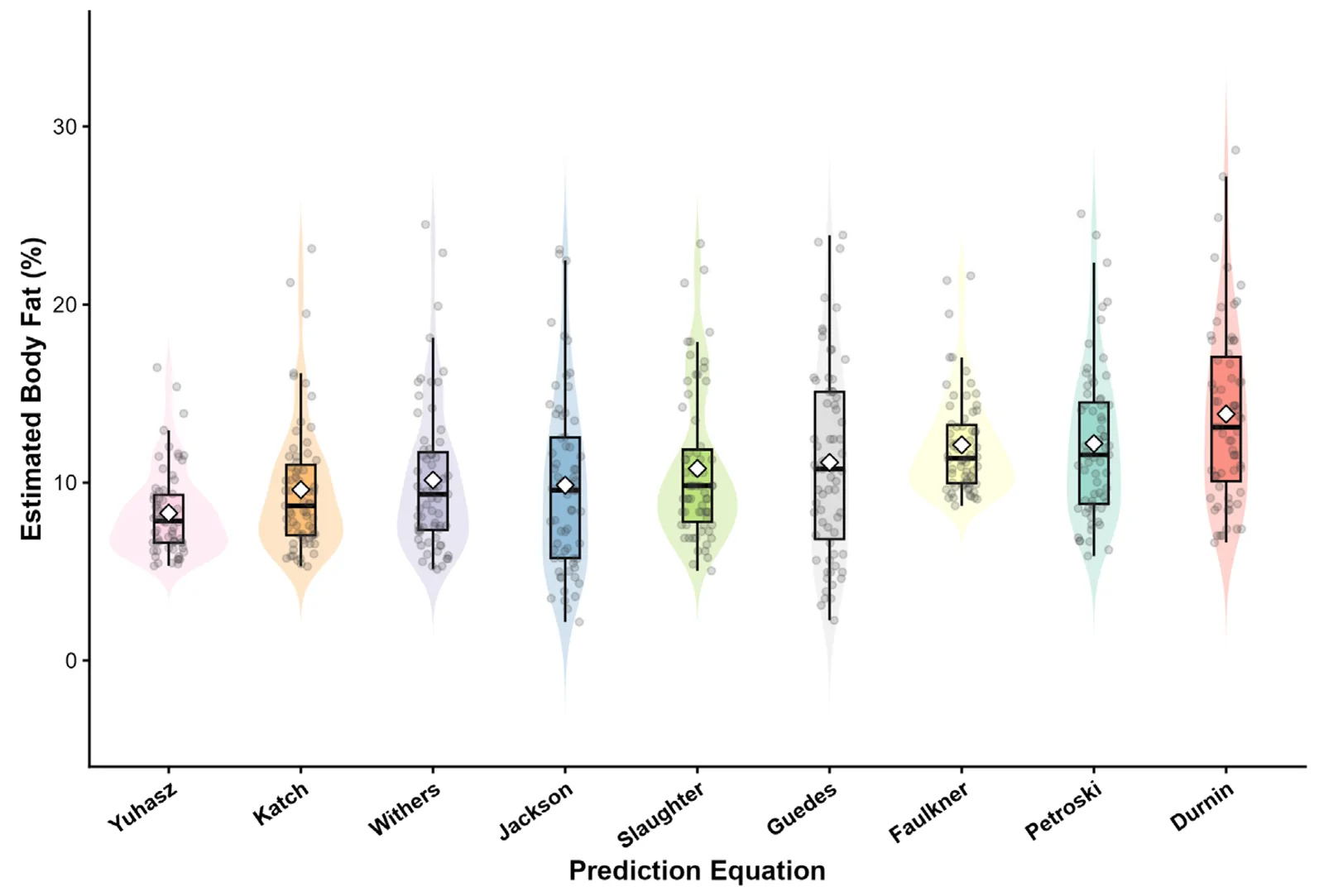
Comparison of estimated body fat percentages across nine anthropometric prediction equations in male combat sports athletes (*N* = 67). Boxplots depict medians (horizontal solid lines) and mean (diamonds), with overlaid violin plots showing data density distributions and individual jittered observations. Significant inter-equation divergence was confirmed via repeated-measures ANOVA.

The repeated-measures analysis confirmed marked systematic divergences across the nine anthropometric equations within the same athletic sample (N = 67; Figure 2). Median relative adiposity spanned a gap of 5.28 percentage points, ranging from 7.84% (IQR: 6.63–9.31%) with Yuhasz [50] to 13.12% (IQR: 10.07–17.06%) with Durnin & Womersley [46], reflecting an average relative disparity of 67.3% in mean estimates between the two extremes (8.28 ± 2.41% vs. 13.85 ± 5.04%). The Katch & McArdle [35] comparator equation yielded a median of 8.69% (9.60 ± 3.69%, IQR: 7.04–11.00%), which aligned closely with athletic-specific equations such as Withers [34] (median: 9.35%, 10.14 ± 4.13%) and Jackson & Pollock [45] (median: 9.57%, 9.85 ± 4.96%). Substantial differences also emerged in distributional dispersion. While Yuhasz [50] (IQR = 2.68%) and Faulkner [48] (IQR = 3.26%, SD = 2.83%) displayed narrow inter-individual variation, general-population models including Guedes [51] (IQR = 8.29%, range: 2.26–23.90%), Durnin & Womersley (IQR = 6.98%, maximum: 28.67%), and Jackson & Pollock [45] (IQR = 6.76%, minimum: 2.17%) exhibited broader variance. These wide limits demonstrate that the choice of prediction equation introduces substantial systematic bias, preventing the interchangeable monitoring of body fat in competitive combat sports.

Bland–Altman agreement analysis and Lin’s Concordance Correlation Coefficient (ρc) against the Katch & McArdle [35] comparator equation demonstrated substantial variability in systematic differences, individual dispersion, and concordance across equations. The Withers equation [34] demonstrated the strongest agreement with Katch & McArdle [35], characterized by a minimal mean bias of +0.54% (SD diff = 1.29%), the narrowest 95% limits of agreement (−1.98% to 3.07%), and substantial concordance (ρc = 0.937, 95% CI: [0.902, 0.959]). Jackson & Pollock [45] similarly displayed a negligible mean bias of +0.26%, though accompanied by wider limits of agreement (−4.19% to 4.71%, ρc = 0.864). Conversely Yuhasz [50] yielded a negative systematic bias (mean bias: −1.31%, 95% LoA: −4.47% to 1.84%, ρc = 0.795), whereas Slaughter et al. [49] (+1.19%, 95% LoA: −2.87% to 5.25%, ρc = 0.826) and Guedes [51] (+1.54%, 95% LoA: −3.05% to 6.12%, ρc = 0.826) exhibited positive systematic differences. Greater systematic discrepancies were observed for Faulkner [48] (mean bias: +2.52%, ρc = 0.735) and Petroski [47] (mean bias: +2.59%, ρc = 0.764), culminating in Durnin & Womersley [46], which produced the highest positive systematic difference (mean bias: +4.25%, upper limit of agreement: +7.89%, ρc = 0.620). Notably, linear regression revealed significant proportional bias across all pairwise comparisons (*p* < 0.05), indicating that inter-method divergence widens progressively at higher levels of adiposity.

**Figure 3:**
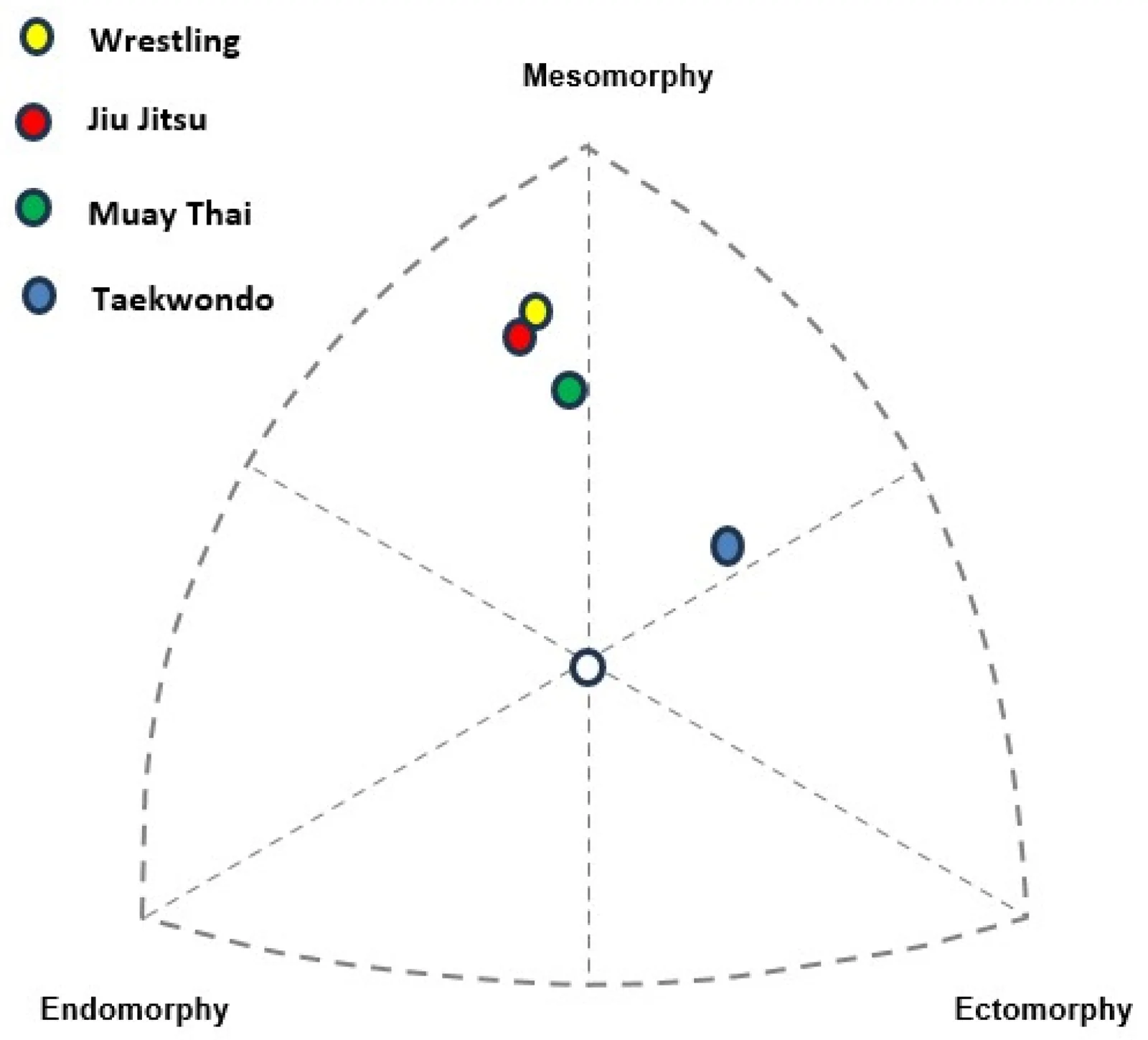
Comparative somatochart of athletes in Jiu-Jitsu, Muay Thai, Wrestling, and Taekwondo.

Martial arts athletes exhibit distinctive differences in BC characteristics according to somatotype, as assessed by the Heath and Carter [42] method (Figure 3). Practitioners of Jiu-Jitsu, Muay Thai, and Wrestling can be classified as endo-mesomorphs, characterized by pronounced musculoskeletal development, with broad skeletal diameters, voluminous musculature, and robust joints (mesomorphic component: 5.0 to 6.5), along with low adiposity and minimal subcutaneous fat (endomorphic component: 1.0 to 2.5). In contrast, Taekwondo athletes are classified as ecto-mesomorphs, exhibiting moderate musculoskeletal development, with muscles, bones, and joints of intermediate thickness (mesomorphic component: 3.0 to 4.5), and a more elongated morphology, with lower body volume per unit of height (ectomorphic component: 3.0 to 4.5). These characteristics reflect specific adaptations to the physical demands of each martial discipline [42].

To examine the multivariate morpho-functional ordination of combat athletes without collinearity distortion, variable reduction from the initial 27-metric matrix to the refined parsimonious PCA model was performed based on statistical correlation thresholds, prior to inspecting group score distributions or discipline separation. The initial 27-variable matrix was dominated by collinear redundant metrics (8 individual skinfolds and 8 overlapping body fat equations), which artificially inflated PC1 variance (64.99%) due to repeated measurement of the same adiposity compartment (inter-variable correlation r > 0.90). To ensure equal domain representation, collinear skinfolds and redundant prediction formulas were removed based on squared cosine contributions (cos^2^ < 0.40) and high correlation thresholds (r > 0.90). The final refined PCA was designed as an explicitly exploratory multivariate ordination technique retaining six non-collinear domain indicators: Arm Muscle Area (regional muscularity), Mesomorphy (musculoskeletal robustness), Ectomorphy (relative linearity), Robustness Index (skeletal frame), Katch & McArdle [35] %BF (athletic adiposity comparator), and Basal Metabolic Rate (metabolic scaling). In this parsimonious model, the first two principal components explained 71.78% of the total variance (PC1: 47.80%; PC2: 23.98%).

Basal Metabolic Rate (BMR) values included in the model were estimated from standardized anthropometric prediction formulas utilizing body mass, height, and body composition variables; although mathematically linked to body mass, BMR was retained to represent the functional metabolic scaling of active tissue, with potential collinearity rigorously controlled through the pre-analysis correlation threshold (*r* > 0.94).

## 4. Discussion

The objectives of this study were to determine the morphological, somatotypic, and proportional differentiation of the Phantom Z-score, to evaluate the degree of systematic and proportional bias in nine anthropometric equations used to predict body fat, and to examine the multivariate morpho-functional ordination of combat athletes using principal component analysis. The main findings of this study show that combat sport is associated with clear differences in the athletes’ morphological structure, proportional musculature, and somatotype, while differences in overall subcutaneous adiposity were less consistent. In taekwondo, it was determined that athletes followed an ecto-mesomorphic profile, characterized by lower relative muscle mass and greater linearity, distinct from the other three sports evaluated (Jiu-Jitsu, Muay Thai, and wrestling).

It is important to acknowledge that chronological age varied significantly across cohorts in the present sample, with Jiu-Jitsu and Muay Thai athletes being older than wrestling and taekwondo competitors. While part of the observed variance in body dimensions and upper-limb muscularity reflects sport-specific technical demands [41,52], chronological age and accumulated athletic career length (training age) also contribute to physiological adaptations, musculoskeletal maturation, and chronic muscular hypertrophy [1,53,54]. More experienced combat athletes typically accumulate years of systematic mechanical loading and resistance training that favor greater muscle cross-sectional area, skeletal robusticity, and mesomorphic dominance compared to younger counterparts [55,56]. Consequently, age-related differences should be considered as a potential confounding factor when interpreting inter-discipline morphological variations [57,58].

### 4.1. Morphological and Somatotypic Differences in Combat Sports

Some previous studies that have aimed to compare body composition among elite athletes in combat sports have revealed that the main differences are related to the specificity of the sport, with the lower extremities showing significant differences in kicking sports (taekwondo, karate, and kickboxing) when compared to other combat sports such as fencing, judo, and wrestling [59]. These results align with those of the present study, which confirmed that grappling and striking disciplines exhibit distinct morphological patterns, suggesting that certain body phenotypes are adapted to the specific demands of each combat sport.

An exploratory review on body composition in combat sports indicated that among male athletes, the predominant somatotype is the endomorphic mesomorph in sports such as jiu-jitsu, wrestling, karate, and judo in the weight categories <90 kg, <100 kg, and >100 kg [60]. These results are consistent with those of the present study, in which elite athletes in jiu-Jitsu, Muay Thai, and wrestling also exhibited the same somatotype in the <80 kg weight category, differing only from the ecto-mesomorphic profile observed in taekwondo athletes in the <70 kg weight category.

Regarding somatotype, the present study determined that mesomorphy had a significant effect on the sport (η^2^ = 0.30), ectomorphy explained 26% of the variance, and endomorphy showed no differences. These results reveal that, in the disciplines evaluated in this study, subcutaneous adiposity is not a distinguishing factor, whereas musculoskeletal development and body linearity are. Furthermore, in this study, the average mesomorphic score was 6.12 ± 0.83 for jiu-jitsu, 5.58 ± 1.17 for Muay Thai, and 5.97 ± 1.44 for wrestling. These values are similar to those reported in studies of other combat sports such as boxing (5.30 ± 0.5), judo (5.30 ± 1.1), and Greco-Roman wrestling (5.7 ± 0.6) [57]. However, reported mesomorphism values for taekwondo athletes in the study by Baranauskas et al. (2024) [57] were 5.7 ± 0.7, and in the study conducted by Yaşar & Sağır (2021) [61], mesomorphism depended on the weight class evaluated (lightweight 6.68 ± 1.49), (middleweight 6.83 ± 1.60), and (heavyweight 8.13 ± 1.05), which differs substantially from the findings of the present study (4.04 ± 1.54). In general terms, the average mesomorphic scores obtained in this study were approximately 5.42, similar to those reported in studies comparing four combat sports, which yielded an average mesomorphic score of 5.4, although, at the same time, these values are low when compared to studies of other Colombian judo athletes with values of 6.24 ± 0.83 [62] and 6.2 ± 1.0 in Brazilian jiu-jitsu athletes [41].

### 4.2. Proportionality and the Use of Equations to Estimate Body Fat Percentage in Combat Sports

The use of Phantom Z-scores adds an element of interest, as it allows for the interpretation of differences by taking body proportionality into account rather than relying solely on absolute values [40]. Even after controlling for differences associated with height, arm circumferences continued to show significant differences among the disciplines evaluated in this study. The sport discipline explained 56% and 55% of the variability in flexed and relaxed arm circumferences, respectively, which supports the interpretation that the observed differences are not exclusively due to certain groups being taller or heavier but may reflect proportionally distinct body configurations and that this variable should be considered in the analysis of athletic performance [63].

The study conducted by Monterrosa-Quintero et al. (2023) [41], which used the phantom Z-score in jiu-jitsu athletes, reported high Z-scores for upper limb circumferences, particularly for the relaxed arm (3.1 ± 1.3 cm) and the flexed arm (3.1 ± 1.2 cm), as well as for the waist (2.2 ± 1.4 cm) values similar to those reported in the present study for the relaxed arm (3.13 ± 1.28 cm), flexed arm (3.12 ± 1.06 cm), and waist (2.26 ± 1.39 cm), which could suggest a distribution of muscle mass. This may be related to another finding of the present study, which showed that the proportional scaling of the phantom model’s Z-score confirmed that upper-body musculature and trunk dimensions differed substantially among combat sports after controlling for height.

In this regard, the use of ISAK phantom Z-scores showed that most anthropometric variables did not differ significantly when analyzing different sports disciplines (individual sports, team sports, combat sports), with significant differences found only for the phantom Z-score of the medial calf skinfold (*p* = 0.013), the phantom Z-score for calf circumference (*p* = 0.022), and the phantom Z-score for humerus width (*p* = 0.026) [58]. This is partly because sport type had its greatest effect on upper limb circumferences, explaining 56% and 55% of the total variance for the Z-scores of the flexed arm and relaxed arm circumferences, respectively (η^2^ = 0.56 and 0.55; *p* < 0.001). These findings suggest that sport-specific demands influence athletes’ body proportions; therefore, further studies are needed to confirm this hypothesis through longitudinal research.

On the other hand, the proportional calf circumference and the eight skinfold measurements showed negligible variation (η^2^ ≤ 0.13) across the four disciplines analyzed, indicating that the relative distribution of subcutaneous fat appears to be homogeneous among these sports after controlling for height. Other studies have reported that representative areas of adipose tissue including the abdominal region and the eight skinfold measurements used to quantify body fat showed lower proportionality than the phantom model reference variable, findings consistent with those described in the literature [41,55].

The pattern of adiposity is, in fact, one of the most methodologically significant findings of the study. Although some equations yielded significant differences between disciplines particularly the equations by Petroski, Jackson, and Guedes, most equations did not identify statistically significant differences. This raises a fundamental consideration regarding the interpretation of differences in adiposity among athletes, which may depend considerably on the estimation method used, and, furthermore, has important practical implications for combat sports, where decisions related to body composition and weight classes can affect nutritional and athletic planning [33]. Therefore, a statistically significant difference obtained using a specific equation does not necessarily represent a real biological difference between groups. These findings are consistent with other studies that have compared team sports with combat sports, reporting that no statistically significant differences in body fat percentage were identified [64], so when estimating body fat percentage, the heterogeneity of body composition must be taken into account [29] in order to interpret anthropometric assessments with reference to the specific characteristics of each sport [58].

### 4.3. Principal Component Analysis in Combat Sports

**Figure 4:**
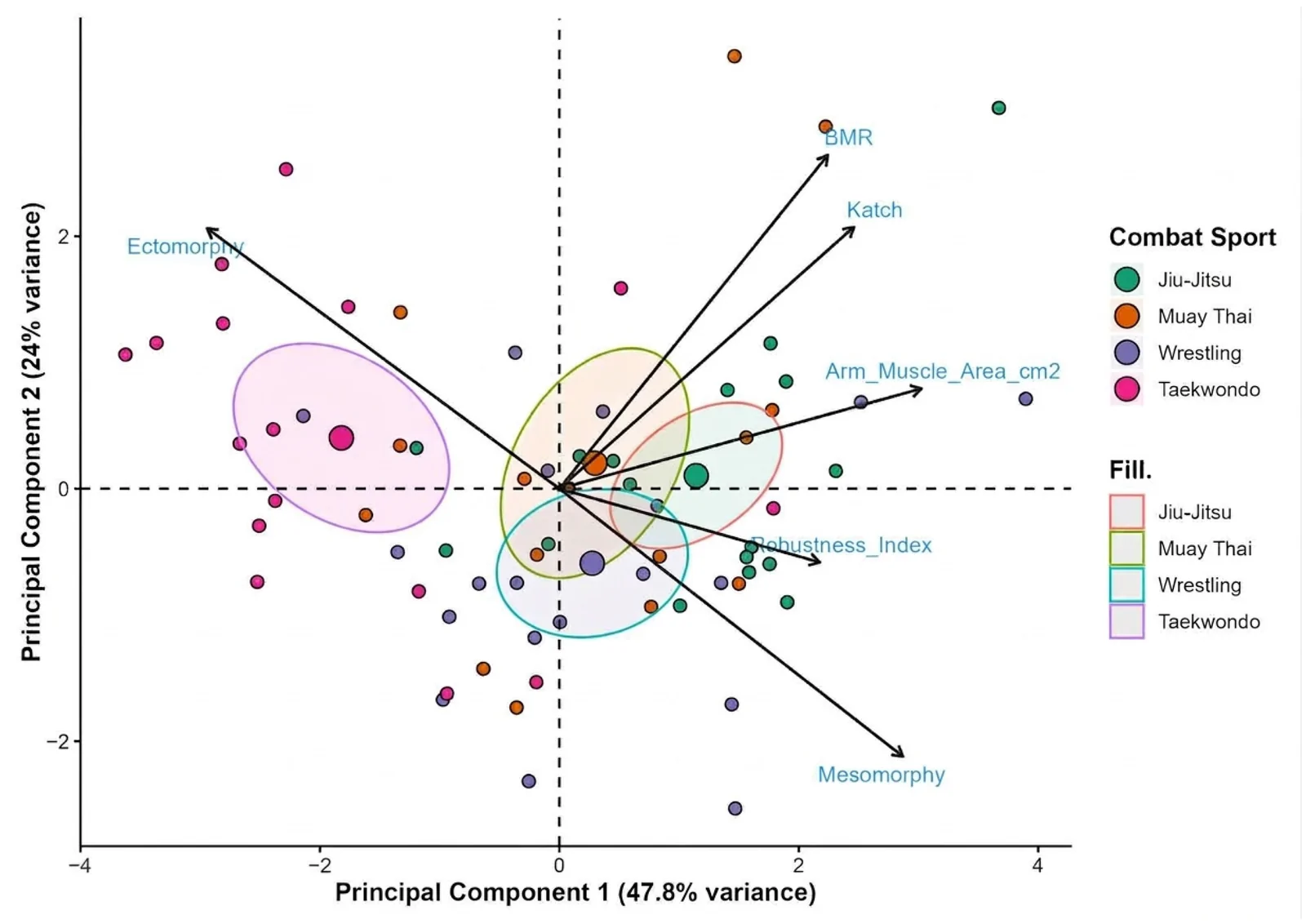
Principal Component Analysis (PCA) biplot depicting the multivariate morpho-functional ordination of male combat sports athletes (N = 67).

Multivariate analysis using PCA (Figure 4) provides a complementary perspective. After removing redundant indicators of body fat, the first two components explained 71.78% of the phenotypic variance. The first component primarily functioned as an axis of robustness versus linearity, with positive loadings for arm muscle area, mesomorphy, body fat estimated using the Katch and McArdle methods [35], basal metabolic rate, and robustness index, and a negative loading for ectomorphy. This structure allowed for a clear differentiation between the more linear morphology typical of taekwondo and the more robust profiles observed particularly in jiu-Jitsu and wrestling. Taken together, these results suggest that the anthropometric profile of combat athletes can be better understood as a multidimensional configuration that integrates muscularity, skeletal robustness, linearity, adiposity, and metabolic characteristics.

Some previous studies that have used PCA in combat sports analyzed the morphometric and somatotypic characteristics of 206 college athletes in jiu-jitsu, judo, karate, taekwondo, freestyle wrestling, and kickboxing, concluding that dimensionality reduction was achieved through three components explaining 68% of the variance, characterized by variables related to body size, height, and upper limb dimensions [52]. Another study of elite taekwondo athletes accounted for 86.54% of the variance based on four principal components involving variables such as height, muscle mass, and, to a lesser extent, muscle percentage and BMI [65]. Thus, it can be concluded that the use of PCA serves to reduce the dimensionality of the data and, thereby, analyze the specific profile of combat athletes [66]. The difference in the amount of variance explained by the dimensions depends on the number of components used to capture the information and on the specificity of each sport [67].

This study, therefore, goes beyond an approach based exclusively on body fat percentage and demonstrates that the morphological characterization of athletes must simultaneously consider different body dimensions. This is particularly important in combat sports, where performance depends on the interaction between body mass, strength production, mobility, speed, and the ability to compete within weight classes. Additionally, it could aid in the design of training protocols and the identification of performance indicators [68].

### 4.4. Limitations, Strengths and Future Directions

This study has several limitations that must be acknowledged. First, the cross-sectional design limits the ability to establish causality between the sports analyzed and the observed body composition characteristics. A second limitation relates to the stratified sample size across the four combat disciplines. Although constrained by active population availability among elite-level cohorts during the evaluation window, sample sizes of 15 to 20 observations per group comply with established statistical rules of thumb for univariate and multivariate group comparisons, ensuring robustness in ANOVA *F*-tests and satisfactory Central Limit Theorem properties. Furthermore, no post-recruitment sample loss occurred, and this sample size provided sufficient statistical power (*β* > 0.80, *α* = 0.05) to detect large effect sizes (*η*^2^ ≥ 0.20). Nonetheless, larger multi-center cohorts would allow for greater precision and enhanced generalizability.

A third limitation stems from the specific inclusion of male athletes, which makes it difficult to extrapolate the findings of this study to female athletes. Furthermore, age could also be a confounding factor; therefore, readers are encouraged to interpret the results of this study with caution. A fourth limitation relates to the use of anthropometric equations to estimate body fat percentage, since no prediction equation in and of itself constitutes a direct measurement of body composition. Accordingly, models such as Katch & McArdle [35] and Withers [34] were interpreted as specialized athletic comparator equations rather than absolute criterion standards. A fifth limitation relates to the use of principal components, which, although they helped reduce the dimensionality of the set of variables, are an exploratory and descriptive tool warrants further investigation. Finally, a sixth limitation relates to measurement reliability metrics; although a single ISAK level 3 instructor conducted all evaluations while adhering to strict duplicate/triplicate measurement criteria, cohort-specific intra-rater technical error of measurement (TEM) values were not systematically recorded for this trial.

Future directions for this research could focus on three categories: the first category associated with different methodological designs, a second linked to the validation of the equations, and a third related to performance indicators in combat sports. Regarding possible methodological designs, longitudinal studies are needed to identify changes in body composition, somatotype, and proportionality in response to different competitive cycles. Likewise, it is important to evaluate the changes that combat athletes undergo during the preseason, specific preparation, and competitive phases. Similarly, it is necessary to conduct multicenter studies to establish differences and similarities among athletes of different ages, athletic levels, countries, and weight classes. This would help confirm whether the patterns identified in isolated studies are consistent across various sporting and cultural contexts. Finally, there is a growing need for controlled trials, crossover studies, randomized controlled trials, and other designs to demonstrate the causal effects that different training approaches and preparation methods have on body composition.

As a second category, studies are needed to externally validate the various body fat percentage equations against reference methods such as dual-energy X-ray absorptiometry (DXA). This would help determine whether the equations used are consistent with one another and, in turn, promote greater accuracy in the assessment of athletes in combat sports. A third category could involve the use of multivariate models or machine learning techniques to help define performance profiles based on morphological variables, physical fitness, technical-tactical performance, and psychological characteristics, among others. This would allow us to move beyond the isolated interpretation of body composition to better understand the performance of athletes in different combat sports.

## 5. Conclusions

The findings of this study demonstrate that there are no similar profiles across different combat sports. Sports such as jiu-jitsu, Muay Thai, and wrestling were characterized by predominantly endo-mesomorphic profiles, while taekwondo exhibited an ecto-mesomorphic profile. These differences were primarily associated with body robustness, upper-body musculature, and body dimensions. This demonstrates that anthropometric assessment in combat sports must take an integrated approach that considers musculature, proportionality, skeletal robustness, somatotype, and the multivariate structure of body composition.

The use of the Phantom *z*-score confirms that the differences persist after accounting for proportionality relative to height, which suggests that distinct morphological specializations exist for the combat disciplines evaluated. Furthermore, the substantial systematic differences observed between anthropometric prediction equations indicate that these equations should not be considered interchangeable when monitoring body-fat estimates in combat athletes. External validation against an independent criterion method (e.g., DXA) is required before recommending a specific equation as the most accurate approach.

## Figures and Tables

**Table 1:** Methodological characteristics of the nine anthropometric body fat prediction equations evaluated in the study.

Reference	Skinfold	Anatomical Sites	Body Fat Method
Katch & McArdle [35]	2	Chest, Subscapular, Thigh (or generalized variants)	Body Density (*Db*) → Siri/Brožek
Withers et al. [34]	7	Triceps, Subscapularis, Biceps, Iliac Crest, Supraspinale, Abdominal, Thigh	Body Density (*Db*) → Siri/Brožek
Jackson & Pollock [45]	3	Chest, Abdomen, Thigh	Body Density (*Db*) → Siri/Brožek
Durnin & Womersley [46]	4	Biceps, Triceps, Subscapularis, Iliac Crest	Body Density (*Db*) → Siri/Brožek
Petroski [47]	4	Subscapular, Triceps, Suprailiac, Calf	Body Density (*Db*) → Siri/Brožek
Faulkner [48]	4	Triceps, Subscapularis, Suprailiac, Abdominal	Direct Body Fat Percentage (%BF)
Slaughter et al. [49]	2	Triceps, Calf	Direct Body Fat Percentage (%BF)
Yuhasz [50]	6	Triceps, Subscapularis, Suprailiac, Abdominal, Thigh, Calf	Direct Body Fat Percentage (%BF)
Guedes [51]	3	Triceps, Suprailiac, Abdominal	Body Density (*Db*) → Siri/Brožek

**Table 2:** Mean and standard deviation values for anthropometric characteristics in martial arts athletes.

Variables	Jiu-Jitsu	Muay Thai	Wrestling	Taekwondo	F (3, 63)	*p*-Value	η^2^
Age (years)	31.9 ± 4.7 ^a,b^	29.4 ± 6.3 ^c,d^	23.3 ± 4.1 ^a,c^	22.8 ± 3.1 ^b,d^	15.90	<0.001	0.43
BM (kg)	81.8 ± 11.6 ^a^	77.8 ± 12.5	71.4 ± 13.2	68.2 ± 10.3 ^a^	4.38	0.007	0.17
Height (cm)	177.2 ± 5.9 ^a^	178.2 ± 8.6 ^b^	170.6 ± 7.2 ^a,b,c^	178.4 ± 5.5 ^c^	5.23	0.003	0.20
**Skinfolds (mm)**							
Triceps	6.6 ± 3.0	7.5 ± 3.6	6.8 ± 2.5	5.6 ± 2.6	1.14	0.338	0.05
Subscapular	11.5 ± 5.4	10.2 ± 4.5	9.6 ± 3.0	8.1 ± 3.1	2.04	0.118	0.09
Biceps	3.0 ± 1.3	3.4 ± 1.8	3.4 ± 1.1	3.0 ± 1.1	0.55	0.647	0.03
Iliac crest	13.3 ± 6.1	14.0 ± 6.5 ^a^	11.2 ± 4.5	8.7 ± 4.3 ^a^	3.08	0.034	0.13
Supraspinale	8.0 ± 3.6	7.7 ± 4.1	6.3 ± 1.9	5.2 ± 2.5	2.74	0.051	0.12
Abdominal	15.9 ± 5.6	15.5 ± 8.9	11.3 ± 5.6	10.3 ± 6.6	2.93	0.040	0.12
Thigh	12.2 ± 4.9	11.8 ± 4.7	9.1 ± 3.8	8.6 ± 4.4	2.93	0.040	0.12
Calf	7.2 ± 2.7	7.2 ± 3.3	6.9 ± 3.5	5.5 ± 3.5	0.95	0.422	0.04
**Girths (cm)**							
Relaxed arm	35.6 ± 3.0 ^a,b^	32.9 ± 3.0 ^c^	31.1 ± 3.0 ^a,d^	27.0 ± 2.7 ^b,c,d^	24.63	<0.001	0.54
Flexed arm	38.2 ± 2.5 ^a,b^	35.7 ± 3.0 ^c^	33.4 ± 3.1 ^a,d^	29.6 ± 2.9 ^b,c,d^	26.30	<0.001	0.56
Waist	85.3 ± 6.3 ^a,b^	81.8 ± 6.5 ^c^	78.3 ± 9.1 ^b^	73.7 ± 4.8 ^a,c^	8.32	<0.001	0.28
Hip	98.8 ± 7.0 ^a^	97.0 ± 6.3	95.6 ± 6.0	92.8 ± 4.9 ^a^	2.85	0.044	0.12
Calf	37.6 ± 2.1	36.6 ± 2.5	36.0 ± 3.1	35.7 ± 2.8	1.76	0.164	0.08
**Breadths (cm)**							
Humerus	7.2 ± 0.4	7.2 ± 0.3	7.1 ± 0.5	6.9 ± 0.3	1.28	0.289	0.06
Femur	9.8 ± 0.5	9.8 ± 0.5	10.0 ± 0.7	9.7 ± 0.5	1.00	0.397	0.05

Note: values are presented as mean ± SD. In terms of Age, significant differences are observed between Jiu-Jitsu and Wrestling (^a^), Jiu-Jitsu and Taekwondo (^b^), Muay Thai and Wrestling (^c^), and Muay Thai and Taekwondo (ᵈ). For Body Mass (BM), a significant difference exists between Jiu-Jitsu and Taekwondo (^a^). For Height, significant differences are found between Jiu-Jitsu and Wrestling (^a^), Muay Thai and Wrestling (^b^), and Wrestling and Taekwondo (^c^). Regarding the Iliac Crest skinfold, a significant difference appears between Muay Thai and Taekwondo (^a^). For Relaxed Arm Girth, significant differences occur between Jiu-Jitsu and Wrestling (^a^), Jiu-Jitsu and Taekwondo (^b^), Muay Thai and Taekwondo (^c^), and Wrestling and Taekwondo (ᵈ). For Flexed Arm Girth, significant differences are noted between Jiu-Jitsu and Wrestling (^a^), Jiu-Jitsu and Taekwondo (^b^), Muay Thai and Taekwondo (^c^), and Wrestling and Taekwondo (ᵈ). For Waist Girth, significant differences appear between Jiu-Jitsu and Taekwondo (^a^), Jiu-Jitsu and Wrestling (^b^), and Muay Thai and Taekwondo (^c^). Finally, for Hip Girth, a significant difference is observed between Jiu-Jitsu and Taekwondo (^a^). All other measured skinfolds, calf girth, and bone breadths show no significant pairwise differences.

**Table 3:** Mean and standard deviation values for Phantom method characteristics in martial arts athletes.

Variables	Jiu-Jitsu	Muay Thai	Wrestling	Taekwondo	F (3, 63)	*p*-Value	η^2^
z-Body Mass	0.9 ± 0.9 ^a^	0.4 ± 0.9 ^b^	0.7 ± 1.1 ^c^	−0.6 ± 1.1 ^a,b,c^	7.56	<0.001	0.26
**z-Skinfolds**							
z-Triceps	−2.0 ± 0.6	−1.9 ± 0.7	−1.9 ± 0.5	−2.2 ± 0.6	1.20	0.318	0.05
z-Subscapular	−1.2 ± 1.0	−1.5 ± 0.8	−1.5 ± 0.6	−1.9 ± 0.6	2.13	0.105	0.09
z-Biceps	−2.6 ± 0.6	−2.4 ± 0.8	−2.3 ± 0.6	−2.6 ± 0.5	0.88	0.458	0.04
z-Iliac crest	−1.4 ± 0.8	−1.3 ± 0.9 ^a^	−1.6 ± 0.7	−2.1 ± 0.6 ^a^	3.16	0.031	0.13
z-Supraspinale	−1.7 ± 0.8	−1.8 ± 0.8	−2.0 ± 0.4	−2.3 ± 0.6	2.67	0.055	0.11
z-Abdominal	−1.3 ± 0.7	−1.3 ± 1.0	−1.8 ± 0.7	−2.0 ± 0.8	2.84	0.045	0.12
z-Thigh	−1.8 ± 0.5	−2.0 ± 0.6	−2.2 ± 0.5	−2.2 ± 0.5	2.28	0.088	0.10
z-Calf	−1.9 ± 0.6	−2.0 ± 0.6	−2.0 ± 0.7	−2.3 ± 0.7	1.01	0.394	0.05
**z-Girths**							
z-Relaxed arm	3.1 ± 1.3 ^a,b,c^	1.9 ± 1.2 ^a,d^	1.8 ± 1.1 ^b,e^	−0.5 ± 1.2 ^c,d,e^	25.28	<0.001	0.55
z-Flexed arm	3.1 ± 1.1 ^a,b^	2.0 ± 1.2 ^c^	1.6 ± 1.2 ^a,d^	−0.5 ± 1.3 ^b,c,d^	26.63	<0.001	0.56
z-Waist	2.3 ± 1.4 ^a^	1.4 ± 1.2 ^b^	1.4 ± 1.7 ^c^	−0.3 ± 1.3 ^a,b,c^	9.41	<0.001	0.31
z-Hip	0.2 ± 1.1 ^a^	−0.3 ± 0.8	0.1 ± 0.9 ^b^	−1.1 ± 1.0 ^a,b^	6.37	<0.001	0.23
z-Calf	0.2 ± 0.7	0.2 ± 1.0	0.1 ± 1.1	−0.5 ± 1.2	1.89	0.140	0.08
**z-Breadths**							
z-Humerus	1.1 ± 0.9	1.0 ± 0.6	1.6 ± 1.1 ^a^	0.3 ± 0.9 ^a^	5.82	0.001	0.22
z-Femur	−0.3 ± 0.8 ^a^	−0.4 ± 0.8 ^b^	0.9 ± 1.2 ^a,b,c^	−0.5 ± 1.0 ^c^	8.33	<0.001	0.28

Note: values are presented as mean ± SD. Superscript letters indicate statistically significant pairwise differences (*p* < 0.05) determined by Tukey’s HSD post-hoc test. In terms of z-Body Mass, significant differences are observed between Jiu-Jitsu and Muay Thai (^a^), Jiu-Jitsu and Taekwondo (^a^), Muay Thai and Taekwondo (^b^), and Wrestling and Taekwondo (^c^). For z-Iliac crest skinfold, a significant difference appears between Muay Thai and Taekwondo (^a^). For z-Relaxed arm girth, significant differences occur between Jiu-Jitsu and Muay Thai (^a^), Jiu-Jitsu and Wrestling (^b^), Jiu-Jitsu and Taekwondo (^c^), Muay Thai and Taekwondo (ᵈ), and Wrestling and Taekwondo (^e^). For z-Flexed arm girth, significant differences are noted between Jiu-Jitsu and Wrestling (^a^), Jiu-Jitsu and Taekwondo (^b^), Muay Thai and Taekwondo (^c^), and Wrestling and Taekwondo (ᵈ). For z-Waist girth, significant differences appear between Jiu-Jitsu and Taekwondo (^a^), Muay Thai and Taekwondo (^b^), and Wrestling and Taekwondo (^c^). For z-Hip girth, significant differences are observed between Jiu-Jitsu and Taekwondo (^a^) and between Wrestling and Taekwondo (^b^). For z-Humerus breadth, a significant difference is found between Wrestling and Taekwondo (^a^). Finally, for z-Femur breadth, significant differences exist between Jiu-Jitsu and Wrestling (^a^), Muay Thai and Wrestling (^b^), and Wrestling and Taekwondo (^c^). All other z-transformed skinfolds, z-calf girth, and z-calf skinfold/girth show no significant pairwise differences.

**Table 4:** Mean and standard deviation values for BC characteristics in martial arts athletes.

Variables	Jiu-Jitsu	Muay Thai	Wrestling	Taekwondo	F (3, 63)	*p*-Value	η^2^
**Somatotype**							
Endomorphy	2.5 ± 1.1	2.4 ± 1.1	2.2 ± 0.8	1.8 ± 1.1	1.39	0.255	0.06
Mesomorphy	6.1 ± 0.8 ^a^	5.6 ± 1.2 ^b^	6.0 ± 1.4^c^	4.0 ± 1.5 ^a,b,c^	9.16	<0.001	0.30
Ectomorphy	1.6 ± 0.8 ^a^	2.2 ± 1.1 ^b^	1.8 ± 1.1^c^	3.4 ± 1.8 ^a,b,c^	7.21	<0.001	0.26
**Body Fat Percentage (%)**							
Petroski [47]	14.2 ± 4.0 ^a^	13.8 ± 4.5 ^b^	11.5 ± 3.6	9.5 ± 3.7 ^a,b^	4.88	0.004	0.19
Faulkner [48]	13.0 ± 2.8	13.0 ± 3.4	11.7 ± 2.2	10.8 ± 2.5	2.52	0.066	0.11
Withers et al. [34]	11.3 ± 4.0	11.2 ± 4.9	9.9 ± 3.6	8.2 ± 3.7	2.00	0.123	0.09
Durnin & Womersley [46]	15.2 ± 4.9	15.3 ± 5.5	13.9 ± 4.5	11.0 ± 4.6	2.78	0.048	0.12
Jackson & Pollock [45]	12.0 ± 4.1 ^a^	11.8 ± 5.4 ^b^	8.9 ± 4.5	6.9 ± 4.3 ^a,b^	4.64	0.005	0.18
Katch & McArdle [35]	10.6 ± 3.7	10.7 ± 4.5	9.2 ± 3.0	8.1 ± 3.3	1.90	0.138	0.08
Slaughter et al. [49]	11.1 ± 3.7	11.8 ± 4.8	11.0 ± 4.1	9.2 ± 4.3	1.13	0.344	0.05
Yuhasz [50]	9.0 ± 2.3	8.9 ± 2.8	8.1 ± 2.1	7.2 ± 2.2	2.21	0.096	0.10
Guedes [51]	13.1 ± 4.4 ^a^	13.1 ± 5.6 ^b^	10.4 ± 5.1	8.0 ± 5.2 ^a,b^	3.71	0.016	0.15
**Σ Skinfolds & Indices**							
Σ 6 Skinfolds (mm)	61.4 ± 21.8	59.9 ± 26.6	52.4 ± 20.3	43.4 ± 20.5	2.22	0.095	0.10
Σ 8 Skinfolds (mm)	77.6 ± 28.4	77.3 ± 34.6	64.6 ± 23.0	55.1 ± 25.7	2.46	0.071	0.10
BMI (kg/m^2^)	26.0 ± 2.8 ^a^	24.4 ± 2.6 ^b^	24.4 ± 3.3^c^	21.4 ± 3.3 ^a,b,c^	6.46	<0.001	0.24
Arm Muscle Area (cm^2^)	90.0 ± 14.3 ^a,b,c^	74.5 ± 11.8 ^a,d^	67.4 ± 12.9 ^b,e^	50.8 ± 8.7 ^c,d,e^	29.40	<0.001	0.58
Arm Adipose Area (cm^2^)	11.5 ± 6.0	12.1 ± 6.5	10.2 ± 4.1	7.5 ± 4.3	2.40	0.076	0.10

Note: values are presented as mean± SD. In terms of Mesomorphy, significant differences are observed between Jiu-Jitsu and Taekwondo (^a^), Muay Thai and Taekwondo (^b^), and Wrestling and Taekwondo (^c^). For Ectomorphy, significant differences are found between Jiu-Jitsu and Taekwondo (^a^), Muay Thai and Taekwondo (^b^), and Wrestling and Taekwondo (^c^). For Body Fat Percentage (Petroski), significant differences occur between Jiu-Jitsu and Taekwondo (^a^) and between Muay Thai and Taekwondo (^b^). For Body Fat Percentage (Jackson & Pollock), significant differences are noted between Jiu-Jitsu and Taekwondo (^a^) and between Muay Thai and Taekwondo (^b^). For Body Fat Percentage (Guedes), significant differences appear between Jiu-Jitsu and Taekwondo (^a^) and between Muay Thai and Taekwondo (^b^). For BMI, significant differences are observed between Jiu-Jitsu and Taekwondo (^a^), Muay Thai and Taekwondo (^b^), and Wrestling and Taekwondo (^c^). For Arm Area, significant differences exist between Jiu-Jitsu and Taekwondo (^a^), Muay Thai and Taekwondo (^b^), and Wrestling and Taekwondo (^c^). For Arm Muscle Area, significant differences occur between Jiu-Jitsu and Muay Thai (^a^), Jiu-Jitsu and Wrestling (^b^), Jiu-Jitsu and Taekwondo (^c^), Muay Thai and Taekwondo (ᵈ), and Wrestling and Taekwondo (^e^). Finally, for BSA, a significant difference is found between Jiu-Jitsu and Wrestling (^a^). All other variables (Endomorphy, remaining body fat percentage formulas, sums of skinfolds and indices, Robustness Index, Arm Adipose Area, BMR, and Energy Expenditure) show no significant pairwise differences. Letters and symbols indicate significant differences between the various martial arts disciplines (*p* < 0.05), as identified by the analysis of variance (ANOVA); BSA = Body Surface Area; BMI = Body Mass Index; BMR = Basal Metabolic Rate.

## Data Availability

The datasets generated and analyzed during the current study are available in Excel format on Figshare and can be accessed via the following DOI: https://doi.org/10.6084/m9.figshare.29852792.
